# P-1163. Drug-Drug Interaction Studies in Healthy Volunteers with BV100 plus Midazolam or Itraconazole as Index CYP3A Substrate and Inhibitor

**DOI:** 10.1093/ofid/ofaf695.1356

**Published:** 2026-01-11

**Authors:** Andrew Shorr, Geza Lakner, Glenn Dale, Lisa Husband, Pierre Delique

**Affiliations:** Medstar Washington Hospital Center; CRU Hungary, Budapest, Budapest, Hungary; BioVersys AG, Basel, Basel-Stadt, Switzerland; BioVersys SAS, Lille, Haute-Normandie, France; BioVersys SAS, Lille, Haute-Normandie, France

## Abstract

**Background:**

BV100 (rifabutin for infusion), developed to treat serious *Acinetobacter baumannii i*nfections, was evaluated in two Phase I, single-center, open-label studies in healthy volunteers to assess for potential pharmacokinetic (PK) interactions with midazolam (a CYP3A4 substrate) and itraconazole (a strong CYP3A4 inhibitor).

**Methods:**

Study 1 (n=23) used a 3-period design: 5 mg oral midazolam on Day 1; BV100 (300 mg intravenous [IV] every 12h, 8 doses total) from Days 3-6; and combination midazolam and BV100 on Day 7. PK parameters of midazolam and 1-OH-midazolam served as primary endpoints.

Study 2 (n=17) used a 2-period design: single dose of 300 mg IV BV100 on Day 1; then from Days 8-21, 200 mg oral itraconazole daily, a dose of BV100 was given on Day 11. Systemic exposure of rifabutin after co-administration with itraconazole was assessed.

**Results:**

In Study 1, BV100 reduced midazolam exposure (C_max_ decreased from 33.36 to 25.43 ng/mL; AUC_₀-tlast_ from 99.20 to 50.81 h·ng/mL). The AUC ratios (test to reference) reflect a decrease in the midazolam AUC of < 50%.

In Study 2, co-administration of BV100 with itraconazole increased rifabutin exposure (C_max_ 1.1-fold, AUC₀-₂₄ 1.3-fold, AUC_₀-inf_ 1.7-fold). These AUC changes were below the threshold defining a moderately sensitive substrate.

BV100 was generally considered safe and well tolerated. No deaths, no serious adverse events (AEs), or other significant AEs occurred. The most frequently reported mild or moderate treatment emergent AEs were infusion site reactions and headaches. One subject (Study 1) had treatment withdrawn due to infusion site reactions possibly related to BV100. We noted no clinically relevant effects of BV100 on clinical laboratory profiles, vital signs, ECGs, physical and neurological examinations, and local tolerability.

**Conclusion:**

BV100 can be classified as a mild (European Medicines Agency) or weak (Food & Drug Administration) inducer of the CYP3A pathway, whereas oral rifabutin is a moderate inducer. The elimination of rifabutin is slightly reduced in the presence of a strong index inhibitor of CYP3A4, resulting in a higher rifabutin plasma concentration for a longer time period. BV100 appears well tolerated. These findings inform future clinical strategies for BV100.

**Disclosures:**

Andrew Shorr, MD, MPH, MBA, Bioversys: Advisor/Consultant Glenn Dale, PhD, BioVersys, SAS: Employee Lisa Husband, MBBS BSc MRCPCH, BioVersys, SAS: Employee Pierre Delique, BioVersys: employee of BioVersys|BioVersys: employee of BioVersys

